# Gender disparities in cognitive impairment across neurological autoimmune disorders: a systematic review

**DOI:** 10.3389/fneur.2025.1555407

**Published:** 2025-04-23

**Authors:** Rahma Ouled Toumi, Chalachew Kassaw, Valeriia Demareva

**Affiliations:** ^1^Department of Cyberpsychology, Lobachevsky State University of Nizhny Novgorod, Nizhny Novgorod, Russia; ^2^Department of Psychiatry, College of Medicine and Health Science, Dilla University, Dilla, Ethiopia

**Keywords:** neurological autoimmune disorders, neuroimmunology, cognition, cognitive impairment, cognitive gender disparities, women’s health, systematic review

## Abstract

**Introduction:**

Neurological autoimmune disorders (NADs) often intertwine with cognitive impairment (CI), representing a multi-layered challenge in both clinical understanding and therapeutic management. The compounded burden of NADs and CI not only significantly affects patient’s quality of Life (QoL), condition’s prognosis, and treatment outcomes, but disproportionately impacts women, who are inherently more susceptible to autoimmunity. This review endeavors to investigate gender-based cognitive deficits, their underlying mechanisms, and their clinical implications. We will focus on Hashimoto’s thyroiditis (HT), Graves’ disease (GD), fibromyalgia (FMS), Guillain-Barré syndrome (GBS), myasthenia gravis (MG), multiple sclerosis (MS), and narcolepsy type 1 (NT1).

**Methods:**

A systematic search of PubMed and the Cochrane Library was conducted for peer-reviewed articles published in the last decade. The search included the keywords “cognitive impairment,” “cognitive decline,” “gender disparities,” “neurological autoimmune disorders,” “Hashimoto’s thyroiditis,” “graves’ disease,” “multiple sclerosis,” “fibromyalgia,” “Guillain-Barre syndrome,” “myasthenia gravis,” and “narcolepsy type 1″. A manual search also took place to uncover grey literature and additional studies we already know exist that did not appear in the two main databases. After applying inclusion and exclusion criteria, 14 articles were selected for analysis. These articles were evaluated for their contribution to unraveling gender-based cognitive impairment trends across NADs and the possible factors involved.

**Results:**

The systematic search yielded a limited number of relevant studies addressing gender disparities in CI across NADs and, apart from MS, most conditions remain under-researched, indicating a significant research gap. While evidence suggests gender-based differences in the manifestations and severity of CI, these findings highlight the necessity for further investigations and innovative clinical approaches tailored to these distinctions.

**Conclusion:**

CI remains a critical, underexplored aspect of NADs, with gender disparities receiving even less attention. Our review highlights a research imbalance and a lack of specific investigations, leading to overgeneralized conclusions about CI across NADs and a limited understanding of the various involved mechanisms. Clinically, addressing CI in NADs requires comprehensive cognitive assessments that account for gender differences, alongside equitable access to resources and personalized treatment approaches. Future advancements are likely to revolve around diagnostic innovations, precision medicine, interdisciplinary collaborations, and holistic approaches to chronic disease management.

## 1 Introduction

Neuroautoimmune disorders (NADs) are a group of autoimmune conditions wherein the immune system’s misdirected response induces neurological impairments alongside organ-specific and systemic autoimmune effects. While the primary targets and pathological mechanisms may differ among these disorders, they share the commonality of progressive immune-mediated damage to neural tissue, disrupting nerve function and signaling, often leading to cognitive impairment (CI), a shared feature manifesting as deficits in various cognitive domains including memory, attention, information processing speed, and executive function. These cognitive impairments not only exert a profound impact on patients’ quality of life (QoL) (1) but also influence therapeutic outcomes, placing substantial strain on global health systems.

Our scope encompasses seven specific disorders; namely, Hashimoto’s thyroiditis (HT), Graves’ Disease (GD), fibromyalgia (FMS), Guillain-Barré Syndrome (GBS), Myasthenia Gravis (MG), Multiple Sclerosis (MS), and Narcolepsy Type 1 (NT1), chosen for their notable global gender-based prevalence and their clinical significance.

Given the multi-factorial nature of these disorders, gender-based mechanisms often play pivotal roles in the prevalence (1), severity (2), and progression of the various clinical manifestations, including CI (3). Biological factors (4), such as hormonal fluctuations, genetic predispositions, and structural mechanisms interact with environmental and psychosocial influences (5), leading to potentially distinct cognitive outcomes between genders. For instance, estrogen’s neuroprotective effects may mitigate CI in women during certain life stages, while postmenopausal changes or male-specific factors may exacerbate the severity of symptoms. Such disparities highlight the intricate interplay within the various neuro-endocrine immune cross-talks involved, further emphasizing the importance of comprehensive patient-centered approaches to mitigate NADs-related CI impacts on both personal and professional patients’ QoL.

Despite growing awareness of these gender disparities in healthcare, there is limited systematic evidence addressing their influence on CI features across NADs. This gap impedes the understanding of autoimmune disorders’ pathophysiological mechanisms and the development of tailored diagnostic and therapeutic strategies. Therefore, this review aims to synthesize available evidence, exploring the prevalence, severity, mechanisms, and clinical implications. By consolidating current knowledge, this study aims to provide a foundation for gender-sensitive care approaches and guide future research directions.

## 2 Methods

### 2.1 Searching strategies

The National Library of Medicine (PubMed) and Cochrane Library databases were searched for eligible studies pertaining to gender disparities in cognitive impairment and neurological autoimmune disorders up to December 26, 2024. We used (“cognitive impairment” OR “cognitive dysfunction” OR “cognitive decline”) keywords combined with the Boolean operator AND for the following keywords (“sex differences” OR “gender differences” OR “gender disparities” OR “male” OR “female”) AND (“neurological autoimmune disorders” OR “multiple sclerosis” OR “Hashimoto’s thyroiditis” OR “Graves’ disease” OR “fibromyalgia” OR “Guillain-Barré syndrome” OR “myasthenia gravis” OR “narcolepsy type1”).

### 2.2 Inclusion criteria

Primary and secondary studies published over the last decade (2013–2024) evaluating gender-based differences of CI manifestations among NADs were included in this study. To ensure strong evidence and direct relevance to our systematic review, we applied filters to include only the most methodologically robust study designs. Clinical trials (Phase II, III, and IV), randomized controlled trials, comparative and controlled clinical studies, pragmatic trials, and multicenter studies were prioritized for their ability to minimize bias and establish causality. Observational studies were included to capture real-world patterns of cognitive disparities across genders. Systematic reviews and meta-analyses were incorporated to synthesize findings from multiple sources, strengthening conclusions. Twin studies were considered for their ability to distinguish gender-related factors from genetic and environmental influences, while validation studies ensured the reliability of cognitive assessments across genders. We also included case reports and datasets while filtering, although considered less robust, as they may highlight rare presentations or enable broad statistical analyses. However, none were retrieved in the final selection, due to the search outcome itself rather than a deliberate exclusion. There were no filters on sex. This approach ensured a comprehensive yet rigorous synthesis, capturing all relevant and methodologically sound studies available on this topic without imposing arbitrary restrictions.

### 2.3 Exclusion criteria

Our exclusion process was conducted in two stages:

#### 2.3.1 Initial screening (database filtering)

During the initial search phase, studies were excluded based on predefined criteria to ensure relevance and feasibility. Studies that were not in English, were not fully and freely available, were conducted on animals, covered age groups younger than 18 years old, or had a broad scope of research that does not align with our main research topic, were excluded. Preprints were also excluded.

#### 2.3.2 Secondary screening (abstract and full-text review)

After retrieving the studies, abstracts and full texts were screened for eligibility based on more detailed exclusion criteria. Studies were excluded if they were irrelevant to the primary research question upon further review, were not accessible despite initial filtering, limiting methodological assessment, had a high risk of bias (RoB), or had missing or insufficient data that hindered meaningful analysis.

### 2.4 Manual search

In addition to the systematic screening of articles based on our predefined inclusion and exclusion criteria, a manual selection of relevant articles was conducted. The Google Scholar database, along with other reliable sources, was searched for secondary references, theoretical evidence, and statistical data, with no restriction applied to the publication date during this phase of the search. This step was crucial to remedy the scarcity of papers identified through the systematic search, enhancing the comprehensiveness and reliability of the findings.

### 2.5 Data extraction and analysis

#### 2.5.1 Study selection process

To ensure methodological rigor, the study selection process followed a structured approach. Initially, the research question and protocol were agreed upon by all authors. Then, the first author conducted the literature search, compiled relevant studies, and shared the primary lists of references with the second and third authors for review. Full-texts of potentially eligible papers were later obtained, reassessed for inclusion during the second screening phase, and a final consensus on the included studies was reached through discussion. Any disagreements at any stage were resolved through consultation with the third author.

#### 2.5.2 Data extraction

Data extraction followed the Cochrane Handbook for Systematic Reviews of Interventions, ensuring a structured and systematic approach. To maintain consistency, the first and second authors collaboratively developed a predefined list of data points in consultation with the senior author. Extracted variables included study characteristics (authors, year, country, study design), population characteristics (sample size, gender distribution, age, clinical status), outcome assessment (cognitive impairment measures, diagnostic tools), predictors/exposures (factors and cofactors, treatment effects, additional variables), statistical analysis methods, main findings, and conclusions. The extracted data were compiled into drafts, iteratively reviewed by all authors to maintain accuracy, before the final version was agreed upon, and presented in a comprehensive table (Table 1).

#### 2.5.3 Quality and bias assessment

To assess the methodological quality of included studies, we evaluated their study design appropriateness, methodological rigor (clear population selection, valid measurement tools, control for confounders, and appropriate statistical methods), handling of missing data, and transparency regarding ethical and financial disclosures.

For risk of bias assessment, we applied validated tools appropriate to each study design. The Newcastle-Ottawa Scale (NOS) was used for simple observational cohort and case–control studies. The ROBINS-I tool was applied to non-randomized studies, including prospective cohort questionnaire studies, observational nationwide cohorts, and cross-sectional studies, for in-depth bias assessments. The JBI Critical Appraisal Checklist was used for cross-sectional studies utilizing questionnaires and mixed retrospective-prospective cohort designs. The SANRA scale was used for the narrative literature reviews, and the Cochrane Risk of Bias 2.0 (ROB2) tool was applied to the only randomized controlled trial (RCT) we were able to retrieve for our research, to assess randomization bias, deviations from intended interventions, missing data, outcome measurement, and selective reporting. Overall, although none of the selected studies exhibited a high risk of bias, we critically ensured that any possible influence on the findings was considered in the discussion.

#### 2.5.4 Data synthesis and analysis

Given the heterogeneity in study designs, populations, cognitive assessment methods, and outcome measures, a narrative synthesis was conducted instead of a meta-analysis. This synthesis followed established guidelines to identify recurring patterns, examine gender-related cognitive differences across NADs, and critically assess the methodological strengths and limitations of the included studies. Where applicable, descriptive statistics, summary tables, and visual representations were incorporated to enhance clarity and highlight key trends.

**Figure 1:**
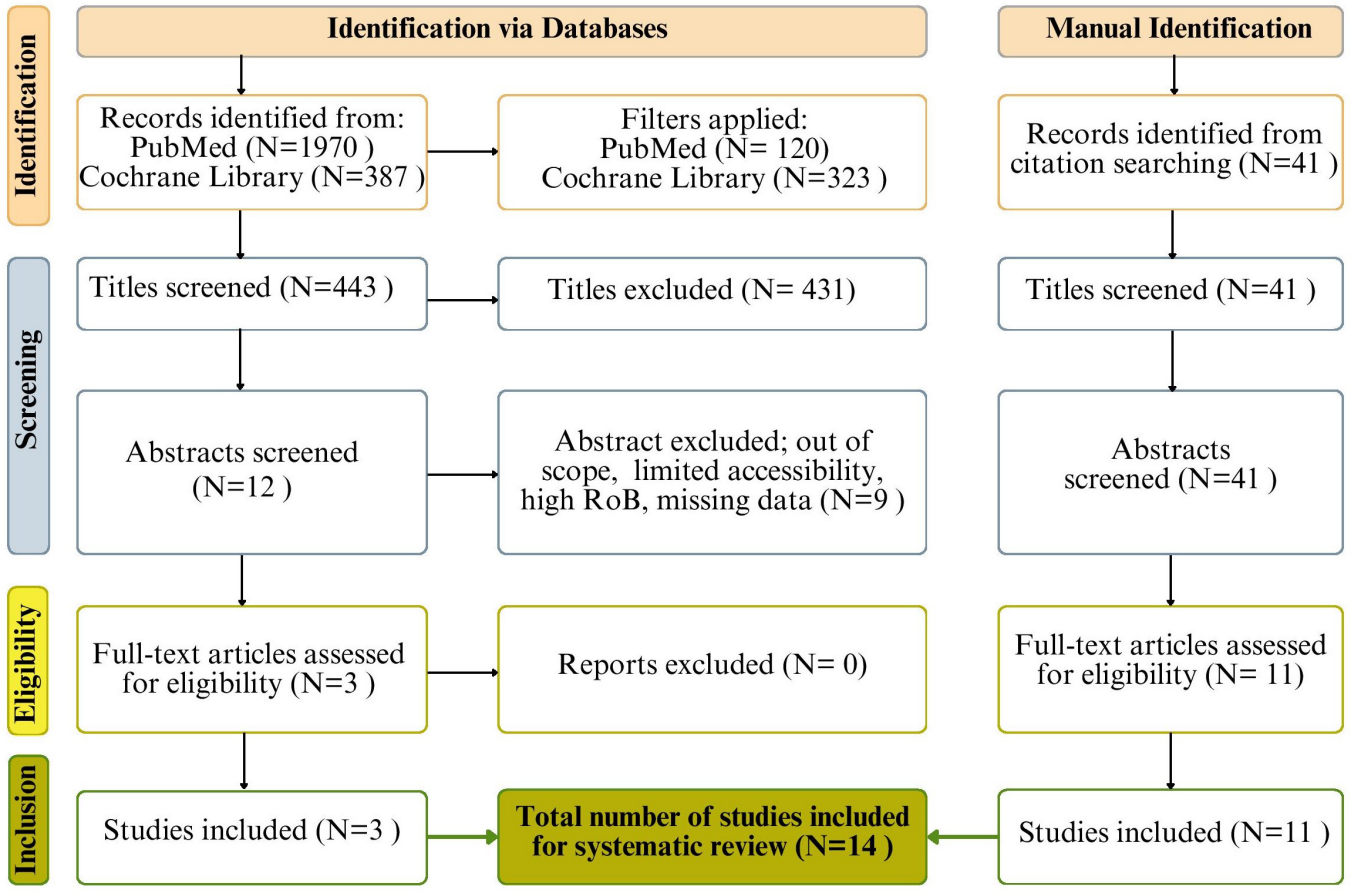
Prisma guideline gender disparities in Cl in NADs.

This review adhered to the Preferred Reporting Items for Systematic Reviews and Meta-Analyses (PRISMA) guidelines. The flow diagram below (Figure 1) outlines the study retrieval process, detailing the number of studies identified, screened for eligibility, assessed for quality, and ultimately included, along with reasons for exclusion at each stage.

#### 2.5.5 Ethical considerations

This review was exempt from ethics approval as it relied exclusively on publicly available, anonymized data sources, ensuring compliance with ethical research standards.

## 3 Results

### 3.1 Overview

Among the seven NADs explored, no studies investigating gender-related differences in CI were found for HT. The number of studies varied significantly among the remaining disorders, with MS receiving the most substantial attention (6 studies), followed by FMS (3), MG (2), and single studies on GD, GBS, and NT1. Importantly, some of the included studies did not directly assess CI but instead focused on QoL. Given the established correlation between QoL and cognitive function, these studies were incorporated as they may offer indirect insights into CI. Their inclusion also helps address the scarcity of available data on gender-related cognitive differences in NADs. These notable gaps may reflect the broader tendency of autoimmune and neurological research to prioritize the physical manifestations of disease and systemic management over cognitive implications, leaving gender-related differences in CI further overlooked. A detailed summary of the findings is provided in Table 1, which is included as a Supplementary material.

### 3.2 Gender disparities in CI among GD patients

The GD search yielded a single prospective cohort study (1) conducted in Sweden, where gender differences were a primary exposure. The study followed 1,186 patients for 6 to 10 years post-treatment to assess disorder-related impacts, and although it does not explicitly address gender-related CI disparities, it was included due to the well-established correlation between psychological health, diminished QoL, and cognitive dysfunction.

The study revealed significant gender disparities in long-term health outcomes. Women, comprising 82% of the study population, reported substantially worse health-related QoL (HRQoL) compared to men. Short-Form Health Status (SF-36) assessments indicated that women experienced higher levels of bodily pain, while Thyroid-Related Patient-Reported Outcome (ThyPRO) measures showed greater depressive symptoms, pronounced impairments in sexual well-being, and increased dissatisfaction with cosmetic outcomes. These findings highlight the potential but unexamined cognitive burden in female GD patients, given the strong link between emotional health and cognitive performance.

Another critical gender disparity was observed in post-treatment investigations, as hypothyroidism following anti-thyroid therapy was twice as prevalent in females as in males (29.5% vs. 14.9%, *p* < 0.05), leading to lifelong levothyroxine dependence. Although the subjective feeling of recovery did not differ significantly between genders, patients requiring levothyroxine (33.2%) reported lower recovery rates compared to non-users (13.9%).

### 3.3 Gender disparities in CI among FMS patients

The three studies on gender-based cognitive disparities in FMS yielded varying findings; revealing both distinct differences and more nuanced or less consistent results.

For instance, the first study (2), assessing symptoms and psychosocial outcomes in 668 FMS patients using a prospective cohort questionnaire design, highlighted that female patients, comprising 90.7% of the study population, exhibited higher tender point counts (TPC) compared to males (*p* < 0.001). However, cognitive dysfunction, symptom severity, and other comorbidities such as depression, anxiety, and sleep disturbances did not manifest through a gender-based pattern. Accordingly, overall OoL was equally impaired in both groups; the association between female sex and lower symptom severity and better OoL, initially validated by the QoL vitality subscale score, was not significant after adjusting for multiple comparisons.

The second study (3), which is a cross-sectional study conducted in Andalusia, Spain on 652 participants, reported that FMS male patients demonstrated better working memory performance compared to their female counterparts according to the Paced Auditory Serial Audition (PASAT; *p* < 0.01), contrary to the healthy control group wherein non-FMS females performed better in memory tasks compared to non-FMS males. Concerning sleep quality, often impaired in this condition, FMS females exhibited reduced sleep latency relative to males. As for symptom severity, male patients exhibited pronounced pain processing deficits compared to the differences seen between FMS and non-FMS females, contradictory to healthy males who demonstrated higher pain thresholds at most tender points compared to healthy females, except at the lateral epicondyle (*p* < 0.01).

Meanwhile, the third study (4), another cross-sectional study conducted on 201 FMS patients and 40 healthy controls in Italy, indicated that females with FMS typically demonstrate higher levels of pain, fatigue, memory challenges, tenderness, balance issues, and sensitivity to environmental stimuli compared to male patients who exhibit a higher frequency of depressive symptoms (72.4% vs. 51.9%, *p* = 0.038). Additionally, the study assessed brain-derived neurotropic factor (BDNF) levels, a protein essential for neurogenesis, synaptic plasticity, and various cognitive functions including learning abilities, memory, and affective regulation. Findings revealed that FMS patients had significantly lower levels compared to healthy controls (*p* < 0.0001). Among all groups, FMS males had the lowest BDNF levels compared to both healthy males and female FMS patients.

### 3.4 Gender disparities in CI among GBS patients

Similar to GD, the GBS search yielded a single study (5) demonstrating persistent psychological and functional challenges among GBS survivors, including depression, elevated levels of anxiety (22% vs. 13% in general population norms), stress, as well as overall impaired personal, social, and professional QoL, long after recovery. This prospective observational cohort, based on data from 76 patients at the Royal Melbourne Hospital, Australia, aimed to identify demographic and clinical factors influencing psychological outcomes and functional independence in GBS. The findings highlighted older age (>57 years), female gender, and prolonged hospitalization (length of stay LOS > 11 days), in line with literature on post-intensive care syndrome (PICS), as key determinants, suggesting a variety of hormonal and sociocultural influences. Accordingly, authors concluded that early psychological interventions are essential to mitigate long-term cognitive and emotional impairments. While cognitive dysfunction was not explicitly discussed, this study indirectly highlights GBS-related CI, as well as the distinguished manifestations of disorder’s burden among different age and gender groups.

### 3.5 Gender disparities in CI among MG patients

The search for gender-related CI manifestations among MG patients identified two relevant studies highlighting key disparities in HRQoL and disease burden. Both studies consistently reported poorer HRQoL among female patients.

The first study (6), which included 1,815 patients from China, revealed that women experienced lower HRQoL across physical, social, and emotional domains (*p* < 0.05), with comorbidities often exerting a more pronounced negative impact. According to the MG activities of daily living (MG-ADL) scale, females had worse scores and higher rates of autoimmune thyroid disease. When navigating related factors, the study emphasized that employment, proactive exacerbation management, and a sustained active lifestyle are crucial for mitigating these challenges (*p* < 0.001).

The second study (7), a mixed retrospective-prospective cohort conducted in Germany, further emphasized these disparities. Females were found to have lower HRQoL, as measured by the Myasthenia gravis-specific 15-item Quality of Life scale (MG-QoL15), and worse functional status (MG-ADL). They also experienced longer delays in diagnosis (2.15 years vs. 0.87 years) compared to males. In terms of comorbidities, depression and overlapping autoimmune disorders were more prevalent among female patients, whereas male patients had higher rates of cardiovascular complications. However, gender disparities did not significantly affect the risk of myasthenic crisis after applying univariate Cox regression analysis (hazard ratio 1.6, *p* = 0.145). As for factors, both higher body mass index (BMI) and greater disease severity, as measured by the Quantitative Myasthenia Gravis Score (QMG), were strong predictors of reduced HRQoL. The study’s authors emphasized the need for lifestyle modifications, gender-specific approaches, and targeted interventions for an optimal MG management.

### 3.6 Gender disparities in CI among MS patients

Consistent with the substantial research focus on MS, CI different manifestations across genders, as well as the possible mechanisms involved, are relatively well acknowledged and have been extensively studied compared to other NADs addressed in this review and beyond. For instance, our search yielded six relevant studies providing interesting insights on the diverse presentation of MS across different subtypes, age groups, and genders.

The first study (8) aimed to investigate whether MS-related CI could be predicted following an initial diagnosis, specifically assessing whether sociodemographic factors such as gender, age, and educational level, along with baseline clinical evaluations and patient’s self-reports, were sufficient to predict the course of progression of MS-related cognitive decline over one-year follow-up. Conducted within the German National Early MS Cohort (KKNMS), the study included 1,123 newly diagnosed patients with MS or clinically isolated syndrome (CIS), with a female-to-male ratio of 2.2:1 and a median age of onset of 31.71 years. The findings revealed that at baseline, 22% of patients exhibited CI, predominantly affecting processing speed and executive function. Male sex was consistently associated with greater CI severity, a trend compounded by other sociodemographic factors such as advanced age, lower educational attainment, significant depressive symptoms, as measured by the Beck’s Depression Inventory Second Edition (BDI-II), and higher physical disability, assessed by the Expanded Disability Status Scale (EDSS). However, the predictive power of these aforementioned demographic factors, as well as conventional clinical assessments, including cranial magnetic resonance imaging (MRI) data and other disease markers remains insufficient for identifying short-term cognitive changes in newly diagnosed MS or CIS patients.

The second study (9), navigating gender-related differences in functional and structural connectivity among MS patients and healthy controls, further illuminate these disparities. Conducted as a cross-sectional case–control study in the United States, the study included 64 participants, comprising 32 MS patients, 16 males and 16 females, and 32 matched healthy controls, with a mean age of 41.85 years. Resting-state functional connectivity analyses revealed that MS patients exhibited significantly stronger connectivity from the posterior cingulate cortex to the medial frontal gyri, anterior cingulate, right putamen, and left middle temporal gyrus compared to healthy controls (*p* < 0.0005), reflecting widespread alterations in neural networks. Notably, sex-specific differences emerged within these patterns. Among healthy controls, males demonstrated stronger connectivity between the posterior cingulate cortex and the left prefrontal cortex compared to females. However, in MS patients, this pattern was reversed, with female MS patients exhibiting stronger connectivity in these regions relative to female controls. Additionally, male MS patients showed significantly weaker connectivity to the caudate nucleus compared to female MS patients (*p* = 0.004), highlighting a potential neural basis for sex-related differences in disease manifestation and progression. These findings emphasize the role of sex as a critical factor in MS-related neural connectivity changes and underscore the need for further research to explore its implications for disease mechanisms and personalized treatment approaches.

The third study (10) consisted of an integrative literature review summarizing existing research on sex differences in MS, with a particular focus on epidemiology, disease progression, hormonal influences, and clinical manifestations. It starts by stating that epidemiologically, MS is more prevalent in females, with relapsing–remitting MS (RRMS) being the predominant phenotype. In contrast, primary progressive MS (PPMS) tends to manifests approximately a decade later and occurs more frequently in males, who also experience worse prognosis, greater CI severity, and heightened disability. Additionally, neuroimaging studies suggest that males exhibit greater gray matter atrophy. Hormonal differences further distinguish disease expression between genders. Male MS patients display lower levels of luteinizing hormone (LH), follicle-stimulating hormone (FSH), and testosterone, with progressive MS males exhibiting more severe hypothalamic–pituitary-thyroid (HPT) axis dysfunction compared to RRMS males. In contrast, the immunomodulatory effects of sex hormones in females are evident in disease activity patterns, as MS symptoms tend to decrease during pregnancy, particularly in the third trimester, only to rebound postpartum and potentially worsen after menopause. Furthermore, adolescent obesity and high BMI have been identified as risk factors for MS development in males. The review also underscores sex-specific comorbidities, with males displaying a higher prevalence of vascular conditions and females being more prone to mental and psychological issues. Authors concluded by emphasizing the need for sex-informed approaches in MS research and medicine, particularly regarding personalized treatment strategies and the safety of disease-modifying therapies (DMTs) during pregnancy and breastfeeding.

Another comprehensive literature review (11), examining the biological contributors to sex differences in MS progression, emphasized that males experience a more rapid disease course, with accelerated central nervous system (CNS) tissue loss and cognitive decline compared to females. Cognitive impairments in male MS patients were particularly pronounced in the verbal and non-verbal memory, information processing speed, attention, and executive functioning domains. Within the same lines, neuroimaging modalities revealed that male patients exhibit lower functional connectivity and reduced network efficiency, which correlated with greater visuospatial memory impairments. Additionally, males demonstrated a higher burden of cortical lesions and were found to be more vulnerable to demyelination, exhibiting diminished remyelination capacities relative to females. In contrast, females showed stronger neuroprotective mechanisms, including more efficient remyelination and a robust antiviral immune response, likely mediated by hormonal influences. The review concluded that these gender disparities in MS-related CI and overall progression stem from a complex interplay of hormonal, immune, structural, environmental, metabolic, and gastrointestinal factors, which interact through intricate and multifaceted biological networks.

In the fifth study, which is an observational cohort investigating the quantitative effects of sex on RRMS activity and disability accumulation (12), researchers demonstrated findings that align with the data of the third study. The study, conducted on a nationwide Danish population of 9,647 patients treated with DMTs since 1996, found that before the age of 45, typically corresponding to the premenopausal phase, female patients exhibited 16% higher relapse rates compared to their male counterparts. However, this difference diminished after the age of 50, suggesting a hormonal influence on disease activity. In contrast, males displayed a steeper trajectory of disability progression, with an annual increase in Expanded Disability Status Scale (EDSS) scores of 0.07 compared to 0.05 in females (*p* = 0.017). Furthermore, males had a significantly higher risk of reaching EDSS milestones, with a 34% greater likelihood of reaching EDSS 4 and a 43% higher likelihood of progressing to EDSS 6 (*p* < 0.001). The findings underscore the robust and sustained immunomodulatory properties of female sex hormones in mitigating disease severity, while highlighting the heightened neurodegenerative burden in males, particularly after midlife.

Accordingly, the only RCT we were able to retrieve (13) further strengthens the previous evidence on female hormones’ immunomodulatory effects. This Phase 2 trial, titled “A Combination Trial of Copaxone plus Estriol in RRMS,” investigated estriol-mediated neuroprotection in 111 female RRMS patients aged 18–50, with 62 receiving estriol treatment and 49 on placebo. Results revealed that estriol treatment significantly preserved gray matter (GM) in the frontal and parietal cortices, correlating with enhanced cognitive performance, as measured by the PASAT. Notably, the estriol group experienced markedly less whole GM atrophy (0.5% vs. 1.5% annualized loss), though no significant effects of estriol on motor outcomes were observed.

### 3.7 Gender disparities in CI among NT1 patients

The only eligible study retrieved from the NT1 search was an integrative literature review (14), synthesizing data from multiple studies to provide a comprehensive understanding of gender disparities in NT1 and other sleep disorders.

The review highlighted that females tend to experience an earlier onset of excessive daytime sleepiness (EDS) and cataplexy, along with more severe symptoms than males. Additionally, women face significantly longer diagnostic delays, ranging from 15.6 to 28 years, compared to 13.8 to 16 years in men, suggesting a combination of clinical and social factors contributing to the disparity. However, conflicting data from some studies indicated no significant gender differences in symptom onset or diagnostic delay. Some studies also pointed to potential self-reporting bias, with women tending to overestimate their symptom severity compared to objective measures. A consistent finding across studies was that male patients encounter fewer nocturnal awakenings than females, who experience more frequent nighttime awakenings, despite longer total sleep time and reduced stage 1 non-rapid eye movement (NREM) sleep. Beyond these sleep-related differences, the review reported potential female-specific NT1 subtypes, such as a mild cataplexy, hallucinations, and sleep paralysis–dominant cluster. Integrating both animal and human data, the paper also acknowledged ongoing research into potential hormonal and neurotransmitter influences, particularly the role of estrogen in sleep–wake cycle regulation and the debated role of hypocretin.

## 4 Discussion

In light of the findings, it is evident that gender-related differences of CI in NADs presents both consistent patterns and notable contradictions within the existing literature. While some similarities across disorders are apparent, significant gaps remain in understanding the full scope of cognitive outcomes, particularly due to small sample sizes, limited generalizability, and underrepresentation of certain age and gender groups. These inconsistencies underscore the urge for breakthrough advances in both research settings and clinical practice. A summarizing table (Table 2) synthesizing these findings and their implications is available as Supplementary material.

### 4.1 HT’s silent toll on female cognition: a research blindspot that need to be addressed

The cognitive sequelae of HT remain critically understudied, especially through a gendered lens, despite its strikingly disproportionate burden on females. HT affects approximately 5% of the global population, making it one of the most prevalent autoimmune conditions worldwide, though its incidence is often grouped within broader thyroid disorder statistics, obscuring its distinct impact, according to the American Thyroid Association. However, current research, predominantly centered on physical manifestations, is overlooking the cognitive consequences that significantly affect patients’ QoL.

The condition manifests through a plethora of neurological and psychological challenges, including mood disturbances, depression, anxiety, memory impairment, reduced concentration, and slowed mental processing. If left untreated or sub optimally managed, thyroid hormone deficiencies may disrupt neural homeostasis, leading to persistent fatigue, emotional lability, chronic stress, and, in severe cases, treatment-resistant depression and neurodegeneration. Hippocampal atrophy and neuroinflammation have been identified as key mechanisms underlying these cognitive deficits, with symptom severity varying widely, ranging from mild to severe symptoms resembling dementia, based on numerous demographic and clinical factors.

Common physical manifestations include myopathy and peripheral neuropathy, resulting in reflex abnormalities, challenges with movement and coordination, and muscle weakness, often misdiagnosed as chronic fatigue syndrome (CFS). Sleep disturbances, such as insomnia, hypersomnia, and obstructive sleep apnea, are prevalent. Other reported issues include hearing impairment and myxedema of the vocal cords. In severe cases, steroid-responsive encephalopathy (SREAT) may occur, manifesting as seizures, transient ischemic attacks (TIAs) or stroke-like episodes, and a spectrum of psychiatric symptoms including hallucinations, and psychosis. These physical impairments further exacerbate CI by increasing emotional lability and physiological strain, underlining the intricate interplay between endocrine stability and neurological resilience.

Compounding these risks, levothyroxine therapy, essential for managing hypothyroidism, has been associated with impairments in memory, executive function, and psychomotor speed, when improperly dosed. Over-replacement can induce a hyperthyroid state, characterized by an increased the metabolic rate, anxiety, irritability, restlessness, hyperactivity, and impaired attention. Additionally, excessive thyroid hormone may disrupt hippocampal function, impairing memory consolidation. Conversely, under-replacement, where thyroid hormone levels are inadequately maintained, results in a hypothyroid state, particularly affecting the hippocampus and prefrontal cortex, marked by significant reduction of the energy available for brain cells to properly process information, retrieve memories, and make decisions. Long-term therapy may also influence neuroinflammatory pathways by stimulating microglial activation, disrupt the integrity of the Blood–Brain Barrier (BBB), and interfere with the balance of neurotransmitters, particularly serotonin, which is crucial for mood regulation and emotional wellbeing.

Along the same lines, emerging research have established the intergenerational neurodevelopmental impact of thyroid dysfunction, with maternal thyroid imbalances linked to an increased incidence of autism spectrum disorder (ASD) in offspring. This underscores the broader implications of thyroid health beyond individual patients, further emphasizing the need for targeted research and intervention.

**Figure 2:**
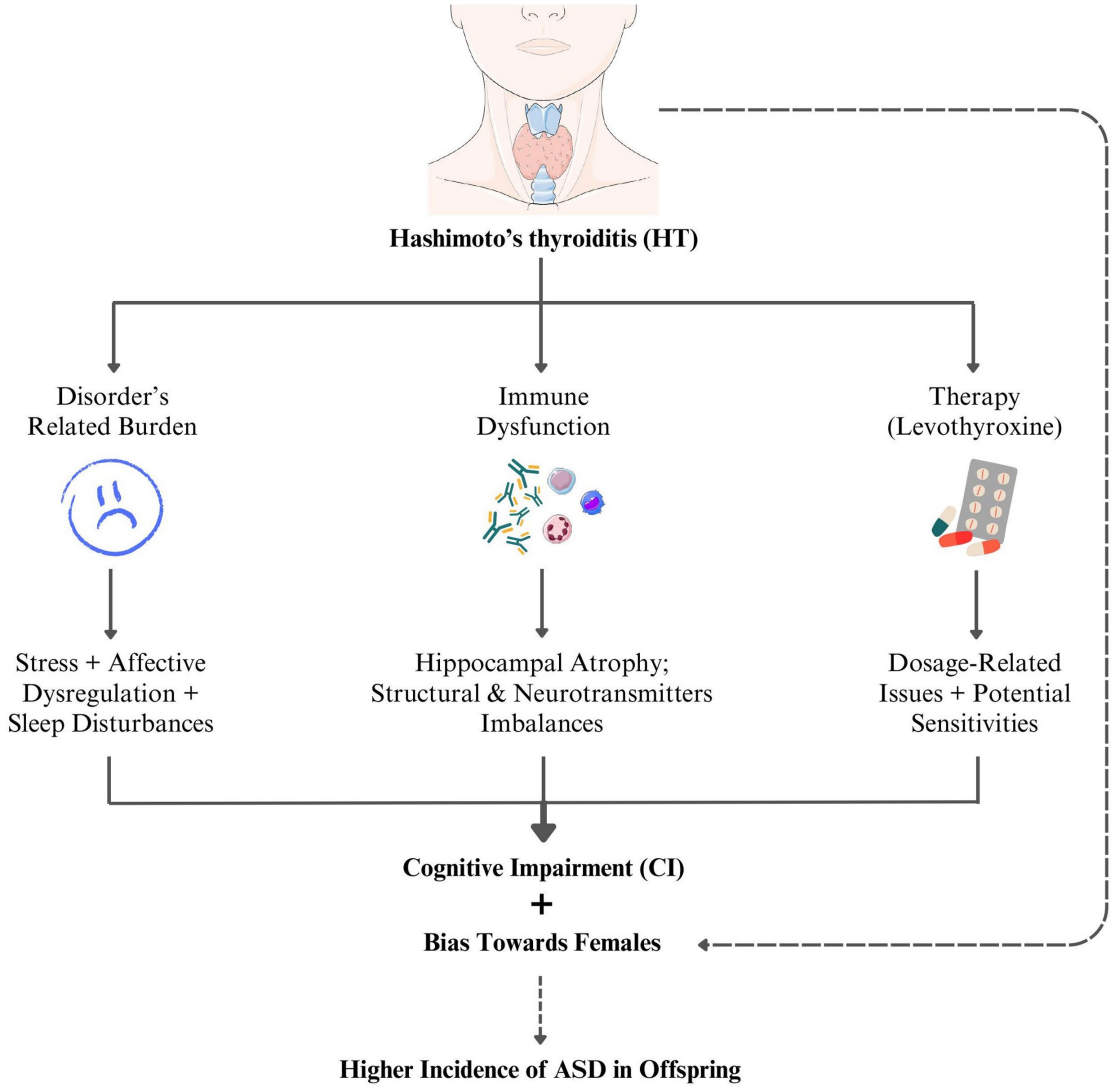
Current landscape of HT-related Cl.

Given the significance of these well-documented correlations, the lack of systematic investigation into CI in HT presents a striking paradox. While research has largely elucidated the factors contributing to women’s heightened susceptibility towards HT, including X chromosome abnormalities, infections, and other environmental triggers, the biological and sociocultural factors modulating the condition’s cognitive impact remain overlooked. Addressing this gap requires prioritizing cognitive preservation as a fundamental pillar of chronic disease care, refining therapeutic strategies to minimize iatrogenic risks, and establish gender-sensitive clinical frameworks. Such advancements would not only improve women’s health but also alleviate broader burdens on global healthcare systems. The following figure (Figure 2) provides an overview of the current landscape of HT-related CI.

### 4.2 GD-related CI disparities: evidence is suggestive but more specific investigations are needed

According to recent data from the American Thyroid Association, GD, the other manifestation of autoimmune thyroid disease (AITD), is recognized as the most common cause of hyperthyroidism in iodine-sufficient areas such as the United States and Europe, with a 0.5 to 1% prevalence and a notable ethnic and gender disparity. Similar to HT, neurological and cognitive complaints are common among patients, with significant variability depending on the course of the disorder, medication regimens, and demographic factors. Ophthalmopathy, also known as thyroid eye disease (TED) and the hallmark feature, often manifests as exophthalmos (protrusion of the eyeballs), diplopia, and restricted eye movement, sometimes extending to tremors. Proximal muscle wasting and atrophy, peripheral neuropathy, and hyperreflexia are also very common. Additional symptoms like heat intolerance, hyperhidrosis (increased sweating), photophobia (light sensitivity), insomnia, and migraines notably affect patients’ social and professional QoL. Neuropsychiatric symptoms include depression, anxiety, irritability, emotional lability, concentration and memory difficulties, and slower cognitive processing. During periods of thyrotoxicosis, these deficits become more frequent. In severe cases associated with untreated or undertreated hyperthyroidism, thyrotoxic crises or thyroid storms may lead to extreme symptoms such as confusion, delirium, seizures, and coma, requiring immediate medical intervention. Accordingly, in clinical practice, experts exercise considerable caution with medication to avoid iatrogenic thyrotoxicosis, a frequent complication associated with hazardous cognitive side effects.

**Figure 3:**
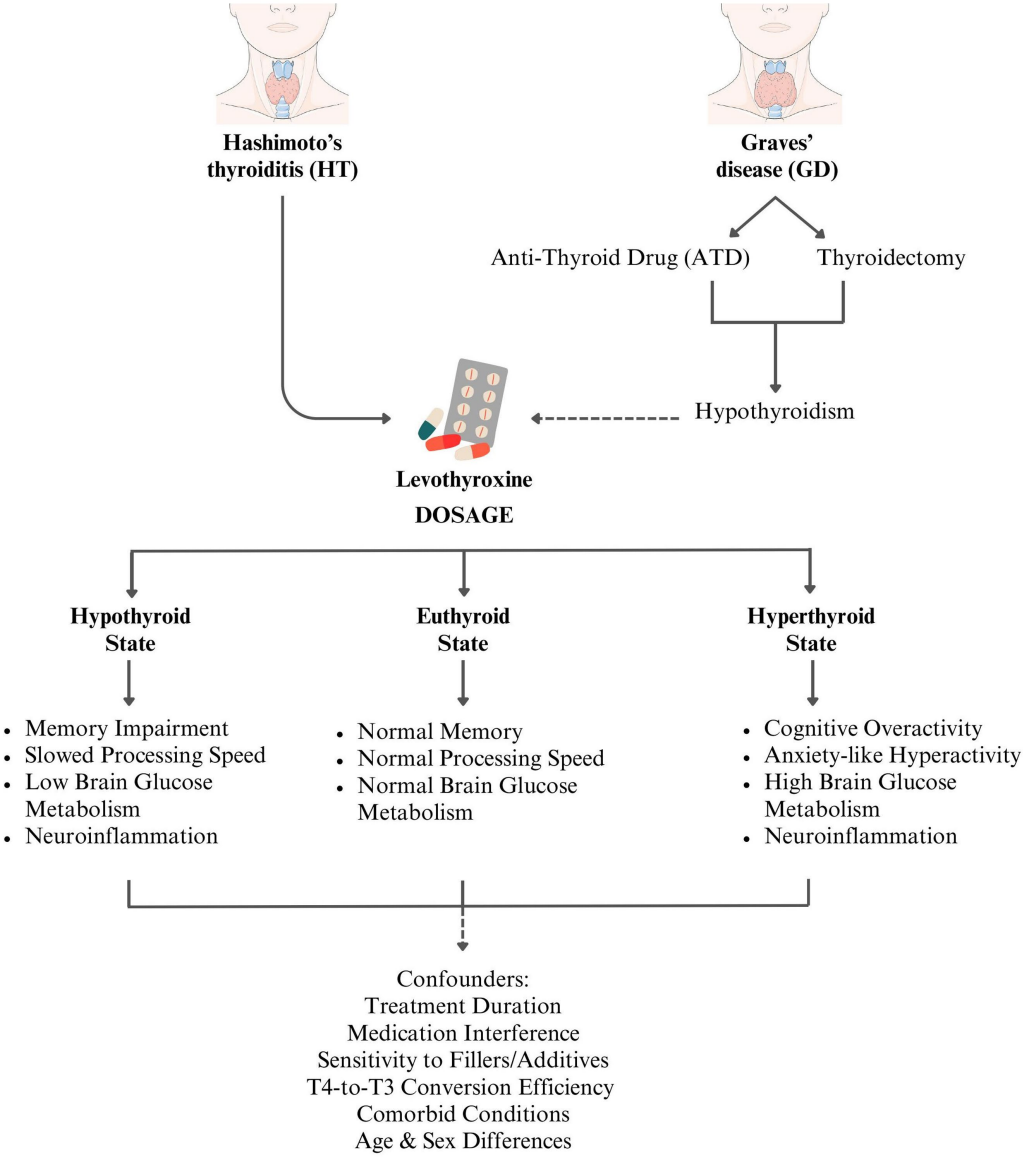
Potential mechanisms of levothyroxine-induced CI in AITD.

Building on this established understanding, our reviewed study (1) highlights critical gender disparities in GD-related cognitive and QoL, particularly in the context of post-treatment outcomes. The heightened depression and bodily pain scores in women with GD align with established literature on AITD’s bias towards females, who often exhibit greater susceptibility to neuropsychiatric symptoms. Several mechanistic pathways may explain these findings. Estrogen, a known modulator of immune function and neuroinflammation, has been implicated in exacerbating autoimmune responses, potentially increasing neuroinflammatory burden in female GD patients. Additionally, dysregulation of the hypothalamic–pituitary–adrenal (HPA) axis, a key component in stress responses, has been more pronounced in women with GD, potentially contributing to the observed higher psychological distress and pain sensitivity. Additionally, alterations in sex hormones and cosmetic concerns, particularly those related to TED, may lower self-esteem and contribute to relationship is satisfaction, further exacerbating psychological distress and ultimately fueling cognitive dysfunction. The significantly higher prevalence of post-anti-thyroid drug (ATD)–induced hypothyroidism in women, necessitating long-term levothyroxine therapy, remains a subject of debate. Proposed mechanisms include sex-based differences in immune response, thyroid gland reserve, and treatment sensitivity, alongside psychosocial factors. Women not only exhibit a greater inherent susceptibility to GD but are also more likely to seek medical care and adhere to long-term treatment protocols. Emerging evidence suggests that levothyroxine itself may be a confounding factor in patients’ perceived recovery and cognitive well-being. Despite achieving biochemical euthyroidism, many patients report persistent fatigue, mood disturbances, and cognitive sluggishness. Potential explanations include interactions between levothyroxine and dietary components or concurrent medications, variability in drug formulations with different excipients that may exacerbate sensitivities or allergies, and the psychological burden of chronic disease management and lifelong medication dependence. The following figure (Figure 3) depicts the potential mechanisms of levothyroxine-induced CI in AITD.

Together, these findings expose significant gaps in both research and medicine. Standard levothyroxine dosing may not adequately account for individual variations in thyroid hormone metabolism, necessiting more personalized approaches, particularly for women. Longitudinal studies are equally needed to unveil whether these gender-related CI disparities stem from underlying disease mechanisms or iatrogenic factors. Comparative studies examining cognitive outcomes in GD patients undergoing ATD therapy versus thyroidectomy could provide valuable insights into treatment-related cognitive risks. Additionally, the lack of significant differences in self-reported recovery between men and women, despite pronounced disparities in clinical outcomes, raises questions about potential symptom underreporting in male patients, an established issue in medical research. This highlights the necessity of more balanced male representation and gender-specific analyses in future studies. Addressing these gaps could advance GD research and clinical management, alleviating challenges for patients, healthcare providers, and global health systems while promoting patient-centered care (PCC) in AITD and beyond.

### 4.3 CI differences in FMS; acknowledged, but inconsistency is calling for more research

According to the National Institute of Arthritis and Musculoskeletal and Skin Diseases, FMS makes one of the most challenging disorders to diagnose due to limited worldwide awareness and variable diagnostic standards and regional healthcare resources. Current data suggests that 2 to 4% of the global population have the condition, with a higher incidence in females. Primarily defined by chronic widespread musculoskeletal pain of moderate to severe intensity, persistent fatigue, and diverse somatic complaints, FMS presents significant neurological and cognitive challenges. Although it remains debated whether these cognitive deficits are primarily disease manifestation or a secondary consequence of its broader systemic effects, recent research has demonstrated a relationship between pain intensity, affective, and cognitive dysfunction. This correlation is based on the overlap of brain areas involved in both pain processing and cognition, leading to the hypothesis that CI in FMS may stem from resource competition within these shared neural networks. Accordingly, patients with FMS typically perform poorly on cognitive tasks requiring management of distractors or competition from multiple stimuli. Hence, key issues include divided attention, concentration difficulties, set-shifting, memory impairments (short-term, working, and semantic), slowed cognitive processing (commonly referred to as “fibro fog”), and sensory abnormalities such as allodynia (pain from non-painful stimuli like light touch or mild pressure) and hyperalgesia (heightened pain sensitivity). Other frequent symptoms include paraesthesia, sleep disturbances such as insomnia and non-restorative sleep, headaches, and orthostatic intolerance. Muscle stiffness and weakness often exacerbate functional limitations. Consequently, mood and psychiatric problems such as depression and anxiety are foreseeable, amplifying the social, personal, and professional challenges experienced by patients. Involved mechanisms include hormonal, immune, metabolic, genetic, and structural factors. Previous studies revealed various dysregulations in vitamin D, cortisol, insulin, growth hormone (GH), and neuro transmitter levels, precisely serotonin, dopamine, norepinephrine, glutamate, gamma-aminobutyric acid (GABA), substance P, and endogenous opioids like endorphins and enkephalins, to name a few.

Accordingly, our systematic research results underscore the complexity of FMS and its heterogeneous clinical presentation. The clear gender disparity in pain-related symptoms, a similar finding across all studies, with women consistently demonstrating pain severity due to higher TPC than men, aligns with the well-documented gender differences in pain perception and response to stimuli, which are thought to be influenced by hormonal, immune, structural, and psychosocial factors, including a potential self-reporting bias (2).

In contrast, CI-related disparities show greater variability in findings related to mood and memory difficulties. Emerging neuroimaging studies suggest that abnormal connectivity patterns may explain outperformance differences in memory tasks between genders and relative to healthy controls. Moreover, recent genetic studies have identified polymorphisms in pain-processing pathway genes that may further contribute to these disparities. Factors such as episodic hormonal shifts, disease duration, and the presence of comorbid psychiatric conditions like depression (4) may also account for inconsistencies across studies, highlighting the need for standardized tools to ensure objective assessments and mitigate confounding effects. Within the same lines, inconsistencies in findings related to sleep disturbances suggest that FMS-related sleep dysfunction does not occur in isolation but likely stems directly from the aforementioned pain perception and psychological stressors. Meanwhile, impaired BDNF levels in FMS patients, as observed in the third FMS study (4), align with emerging molecular data linking symptom improvement to the downregulation of salt-inducible kinase 1 (SIK1). This serine/threonine kinase plays a key role in in energy metabolism, stress response, and inflammation, and is significantly involved in pain perception pathways. Notably, SIK1 is a crucial co-factor in BDNF regulation, suggesting a potential interaction that may underlie the pathophysiology of FMS. Given its role, SIK1 has recently emerged as a promising biomarker and therapeutic target within the framework of precision molecular therapy (MT), pending validation in larger and more diverse cohorts. Meanwhile, its complex interaction with female hormones is still under investigation.

Therefore, future therapeutic strategies should integrate these molecular insights into more comprehensive treatment protocols. Existing approaches, such as restoring N-acetyl-aspartate (NAA) levels, have already demonstrated efficacy in normalizing glutamate levels and modulating myo-inositol, a marker of glial activation. From a research perspective, future studies should prioritize comprehensive gender-specific, objective assessments using validated tools and pre-defined protocols. Additionally, increasing male representation in FMS research, particularly through longitudinal study designs, is essential for capturing symptom progression over time and refining diagnostic and therapeutic approaches, accordingly.

### 4.4 GBS and cognitive health; CI is often a secondary concern, making gender-related cognitive disparities even more overlooked

According to the Centers for Disease Control and Prevention, GBS is a relatively rare disorder, with an incidence of approximately 1 to 2 cases per 100,000 individuals annually. Notably, the number of cases tends to surge during viral outbreaks, as observed with the Zika virus and the COVID-19 pandemic. The condition often manifests with a higher prevalence in males and is characterized by acute inflammatory demyelination of peripheral nerves, typically presenting with rapid onset of muscle weakness beginning in the legs and progressing upwards, potentially leading to various issues such as respiratory failure and movement difficulties that may evolve into total paralysis. This ascending muscle atrophy is often accompanied by paraesthesia of variable intensity. Within the same lines, the loss of reflexes is a typical hallmark sign and a key diagnostic feature distinguishing GBS from other neurological conditions. Autonomic dysfunction is also common, manifesting as blood pressure dysregulation, heart rate (HR) abnormalities, and bladder and bowel dysfunction. In rare cases, if the cranial nerves are involved, dysphagia (difficulty swallowing), dysarthria (difficulty speaking), and diplopia (double vision) may occur.

While the primary focus of GBS research has been on motor and autonomic dysfunctions, accumulating evidence suggests that cognitive and psychological complications, though often underrecognized, significantly impact long-term patient outcomes. Our systematic search aligns with broader findings indicating that cognitive impairments are particularly evident in the acute inflammatory phase, especially among patients requiring prolonged hospitalization or intensive care unit (ICU) admission. High-risk subgroups include females, older adults, and those undergoing extended hospitalization (5). Delirium, post-intensive care syndrome (PICS), attention deficits, short-term memory impairments, and executive challenges with planning, problem-solving, and organization are frequently reported. These complications are further exacerbated by comorbid factors such as chronic stress, sleep disturbances, reduced energy levels, affective dysregulation, and the neurocognitive side effects of GBS medications. Common treatments, including corticosteroids, intravenous immunoglobulin (IVIG), and plasma exchange, may impair cognitive integrity through various mechanisms such as corticosteroid-induced hippocampal dysfunction, IVIG-related neuroinflammatory responses, and metabolic disruptions. Psychological burdens, including depression and anxiety, not only influence cognitive function but also contribute to social difficulties and interfere with rehabilitation efforts.

Despite these observations, gender-specific differences in GBS-related cognitive and emotional burdens remain largely unexplored. Current research is limited by several methodological constraints, including data scarcity, small sample sizes that poorly represent the GBS community, and an indirect assessment of cognitive dysfunction, which is often inferred from psychological correlations rather than direct neurocognitive evaluations. Addressing these gaps requires longitudinal studies with standardized cognitive assessments to accurately characterize the cognitive profile of GBS patients and its underlying mechanisms. Future research should also further explore the interplay between neuroinflammation, autonomic dysfunction, and CI. Given that inflammatory cytokines, BBB disruption, and peripheral immune activation are implicated in other autoimmune disorders with cognitive components, similar pathways may contribute to GBS-related cognitive deficits. Integrating these mechanistic insights with targeted therapeutic strategies could pave the way for improved long-term management and individualized treatment approaches.

### 4.5 MG-related CI and females’ poorer long-term outcomes; current data points to chronic fatigue

The Myasthenia Gravis Foundation of America states that MG prevalence remains difficult to determine due to diagnostic limitations in certain regions, likely leading to underreporting. However, current estimates suggest that MG affects approximately 700,000 individuals worldwide, with an approximate prevalence rate of 10 to 20 cases per 100,000 people. The disorder manifests with a fluctuating muscle weakness, which typically worsens with exertion and improves with rest, making chronic fatigue a hallmark feature. Ocular muscle involvement is often the initial symptom, while bulbar muscle weakness may lead to dysphagia, dysarthria, and difficulties with facial expressions and mastication. Involvement of proximal limb and neck muscles can result in movement restrictions and dropped head syndrome, respectively, while phonatory muscle exhaustion often manifests as a soft, nasal, or monotone voice. In severe cases, respiratory muscle compromise can lead to respiratory insufficiency or failure, a medical emergency known as myasthenic crisis.

Beyond the primary neuromuscular manifestations, MG, similar to GBS, may indirectly lead to CI, particularly in the domains of short-term and immediate memory, visuospatial processing, verbal fluency, and information processing speed. Mental fatigue, a prominent feature, is thought to contribute significantly to these deficits, alongside the burden of persistent sleep disturbances, including insomnia and sleep apnea. Psychophysiological symptoms such as depression and anxiety further exacerbate disease burden, reducing HRQoL and complicating disease management.

Emerging evidence highlights that female sex has been identified as a significant predictor of reduced HRQoL, with studies indicating that women with MG report greater impairments in daily functioning and psychosocial well-being compared to their male counterparts (6). This disparity appear to be mediated by multiple factors, including higher rates of comorbidities, employment status, and the frequency of disease exacerbations. Notably, the impact of BMI has emerged as a point of inconsistency across studies, with some research identifying higher BMI as an independent predictor of reduced HRQoL (7). The rationale behind this correlation is that elevated BMI-induced CI manifests through interconnected metabolic, inflammatory, vascular, and hormonal pathways. Excess adipose tissue promotes chronic low-grade inflammation, with pro-inflammatory cytokines like Tumor necrosis factor alpha (TNF-*α*) and interleukin 6 (IL-6) activating microglia, disrupting BBB integrity, and impairing synaptic plasticity. Obesity-induced insulin resistance further compromises cognitive function by reducing insulin transport into the brain, impairing glucose metabolism, and promoting amyloid accumulation. Additionally, cerebrovascular dysfunction, driven by hypertension and endothelial impairment, limits cerebral perfusion and increases the risk of silent strokes. Hormonal imbalances, including leptin resistance, cortisol dysregulation, and estrogen depletion in females, further exacerbate cognitive decline. All the while, sleep disturbances, as previously mentioned, particularly obstructive sleep apnea, contribute to intermittent hypoxia, oxidative stress, and impaired memory consolidation. Additionally, gut dysbiosis associated with obesity influences neuroinflammation and neurotransmitter synthesis, further linking metabolic health to cognitive outcomes. Given these mechanisms, weight management is a critical point that needs further investigations; while some consider it an essential component of MG care, others see that targeted metabolic interventions do not directly mitigate MG-related CI but are rather correlated with psychological wellbeing.

Overall, current research remains limited in scope. Further research of the mechanistic links between MG, fatigue-related CI, and gender-specific vulnerabilities are needed. Acetylcholine signaling and disruptions in cholinergic pathways, particularly within the CNS, and their indirect contribution to CI through persistent fatigue, are also another interesting research avenue. Additionally, interactions between MG pathophysiology and neuroendocrine factors, such as estrogen and cortisol fluctuations, require more in-depth investigations. Medically, personalized interventions addressing comorbidities, psychosocial support, and lifestyle modifications, such as tailored exercise programs and dietary strategies to optimize weight management and mitigate inflammation, as well as gender-specific strategies to alleviate the pronounced disturbances reported by female patients with daily living activities, may play a pivotal role in improving outcomes, while research in this field advances.

### 4.6 MS; CI is a primary concern, and gender-related cognitive differences are relatively well explored

According to the Multiple Sclerosis International Federation, MS, one of the most extensively studied NADs, effects around 2.88 million people globally with a prevalence of about 0.036%, (approximately 36 cases per 100,000 people) mostly in regions far from the equator such as North America and Europe, likely due to environmental and epigenetic factors including sunlight exposure and vitamin D levels. This extensive research focus can be attributed to MS’s highly heterogeneous nature, both in its clinical presentation and progression, as well as its observed correlations with other neurodegenerative disorders burdening the global healthcare system, such as Alzheimer’s disease and dementia.

MS is characterized by central nervous system demyelination, causing symptoms such as chronic fatigue, asymmetrical muscle weakness, spasticity, ataxia (impaired coordination and balance), paraesthesia, and neuropathic pain. Visual impairments, including nystagmus and optic neuritis, may cause various complications ranging from blurred vision and altered color perception to blindness. Cognitive deficits are pronounced, including visual, verbal, and working memory impairment, slower information processing, attention deficits, visuospatial problems, and executive function difficulties, especially with tasks requiring problem-solving, decision-making, and multitasking skills. Social cognition, such as emotion recognition and theory of mind (ToM), is also impaired, especially in progressive MS subtypes like PPMS. Additionally, speech and swallowing difficulties, bladder and bowel dysfunction, autonomic issues, sleep disturbances, and psychological challenges such as depression, anxiety, and irritability are often reported.

The severity of cognitive deficits vary across the various demographic groups and MS clinical subtypes; RRMS is generally characterized by milder CI, SPMS often exhibits more severe and widespread deficits, while PPMS predominately features persistent challenges with processing speed and executive functions. Current evidence suggests that MS-related CI is the outcome of a complex interplay between structural, immune, hormonal, and metabolic factors. Structural changes, including both cortical and deep GM structures atrophy and volume reduction, white matter lesions, and widespread disrupted connectivity within critical brain networks such as the default mode network (DMN), explain the various challenges MS patients experience with memory, processing speed, and executive function. Microstructural abnormalities within the Yakovlev’s basolateral limbic circuit, a neural pathway connecting the orbitofrontal cortex, thalamus, amygdala, and hippocampus and regulating both affective and cognitive processing, demonstrated altered radial diffusivity and fractional anisotropy in these tracts. Such alterations further highlight the previously mentioned link between MS and progressive neurodegeneration.

Hormonal dysregulations, mainly involving sex hormones, vitamin D, insulin, and cortisol, significantly influence MS pathology and cognitive outcomes. For instance, estrogen and progesterone are often impaired. Given their crucial roles in neuroprotection and synaptic plasticity, fluctuations in these hormones during phases of hormonal shifts such as the menstrual cycle, pregnancy, postpartum, and menopause contribute to variations in cognitive burden among female MS patients. Estrogen promotes remyelination by enhancing oligodendrocyte precursor cell differentiation and supports axonal stability, while both estrogen and progesterone regulate neuro inflammation by modulating cytokine release and T-cell activity. Additionally, estrogen influences microglial function by promoting the anti-inflammatory M2 phenotype, thereby reducing neurotoxic effects. A key mechanism underlying its neuroprotective role is the inhibition of the NF-κB pathway, which prevents the transcription of pro-inflammatory cytokines such as TNF-*α*, interleukin-1 beta (IL-1β), and IL-6. Studies have shown that estradiol therapy downregulates these markers, correlating with reduced neuroinflammation and improved cognitive performance in MS models. Furthermore, estrogen’s antioxidative properties help counteract oxidative stress, a key driver of neurodegeneration.

In males, who are two to three times less likely to develop MS than females, estrogen is primarily synthesized through a process called testosterone aromatization. Research demonstrates that the conversion of testosterone to estradiol improves verbal memory by binding to estrogen receptors in key brain regions such as the hippocampus and prefrontal cortex, promoting synaptic plasticity, neurogenesis, cholinergic activity, and dopamine signaling, all of which are crucial to language processing and recall. Meanwhile, spatial memory improvements appear independent of this conversion.

Interestingly, despite MS’s pronounced prevalence in women, studies show that men exhibit more severe MS-related CI symptoms. This disparity may be attributed to lower testosterone levels, the stronger immunomodulatory effects of female sex hormones, and the weaker neural compensatory mechanisms observed in males.

Another factor that may hold the answer concerning the higher frequency of relapses often experienced by female patients is vitamin D deficiency, a hallmark of most autoimmune disorders. Vitamin D plays essential roles in calcium signaling, neuroprotection, immune function, and numerous chemical and metabolic regulations, making its deficiency a significant contributor to MS pathophysiology. In contrast, the more severe symptoms reported in males may be explained by other compounding metabolic factors such as insulin resistance, frequently correlated with sedentary lifestyle habits, including smoking, alcohol consumption, and substance abuse, all of which are more prevalent in men than women.

Within the context of immune-mediated mechanisms, demyelination, central to the pathology and progression of MS, is mostly initiated by B-cells, which damage myelin and oligodendrocytes through multiple pathways, including the production of myelin oligodendrocyte glycoprotein and myelin basic protein production, T-cells activation, oligoclonal bands secretion, and amplification of other inflammatory cascades. According to aforementioned findings of our systematic search, the increased vulnerability of males to demyelination and their reduced neural repairing capacities warrants further explorations of the various factors involved, with a special attention to female sex hormones involvement, in order to unravel the underpinnings of such disparity.

### 4.7 NT1, NT1-related CI, and gender-related CI disparities among narcoleptic patients; three under-explored areas

According to the National Institute of Neurological Disorders and Stroke, NT1, although often underdiagnosed due to symptoms overlap with other sleep disorders, affects approximately 4 million people worldwide, with a prevalence of 0.05% (around 1 in 2000 individuals), often influenced by both environmental and genetic factors. The condition is characterized by the dysregulation of the sleep–wake cycle, sleep paralysis and severe drowsiness during inappropriate times such as conversations, eating, and driving. Nocturnal sleep is typically fragmented, with rapid transitions into rapid eye movement (REM) sleep. Hypnagogic and hypnopompic visual, auditory, and tactile hallucinations are common, alongside automatic behaviors where patients engage in routine activities without full awareness or memory of their actions. Cataplexy, triggered by strong emotions, may also occur, ranging from slight issues with eyelids or jaw weakness to complete muscle collapse. Cognitive challenges, including difficulties with short-term memory, information retention, and attention, likely arise from widespread hypocretinergic projections to the cortex, hippocampus, and limbic regions. These cognitive symptoms are exacerbated by chronic sleep fragmentation and dysregulation of orexin-mediated neurotransmission, which in turn affects dopaminergic and noradrenergic pathways critical for arousal and cognition. Additionally, inflammation-driven neurodegeneration is an emerging factor, with cerebrospinal fluid (CSF) analyses revealing altered cytokine profiles, increased BBB permeability, and T-cell-mediated autoimmunity against hypocretin-producing neurons.

Despite the pronounced impacts of these impairments on both personal and professional performance, as well as the increased rates of mood disorders and emotional dysregulation among NT1 patients, CI remain poorly defined, especially in the context of gender-dependent symptomology. While some studies suggest potential hormonal modulation of hypocretinergic activity, particularly through estrogen’s neuroprotective effects, the lack of standardized neuropsychological assessments and sex-stratified analyses limits definitive conclusions. Given the complexity of NT1 pathophysiology, further mechanistic research is needed to delineate the interplay between neuroendocrine, immune, and neurotransmitter systems in NT1-related CI. Future studies should also consider confounding factors such as disease duration, lifestyle, comorbidities, and pharmacological treatments to refine our understanding of NT1’s heterogeneous cognitive and neuropsychiatric manifestations.

## 5 Conclusion

In conclusion, the clinical features of NADs, including cognitive deficits, manifest with significant variability in intensity and progression trajectories. This variability poses substantial challenges to research organizations, healthcare systems, economies, and societies due to the interplay of complex mechanisms, the potential for severe disability, increased risk of related accidents, and the need for prolonged care. Addressing these challenges becomes even more demanding when considering gender-based disparities.

Our systematic review, although highlighted evidence of gender-based CI differences, encountered several limitations, including a scarcity of relevant studies, unequal research attention across different disorders, and a lack of standardized methodologies, all of which which hinder cross-study comparisons and robust conclusions. Additionally, research gaps persist regarding the impact of pharmacological treatments on cognition, the role of overlapping comorbidities, and the influence of neuroendocrine-immune interactions in shaping cognitive outcomes. The underrepresentation of diverse age groups, ethnicities, and specific populations, such as pregnant individuals and people with overlapping comorbidities, further limits the generalizability of current findings.

To address these challenges, future efforts should prioritize global awareness of CI in NADs and advocate for health policies that enhance accessibility to optimal diagnostic modalities. Equally crucial is the need for comprehensive and equitable investigations of various NADs and their potential interrelationships, ensuring adequate representation of diverse populations. Given the inherent subjectivity of patient-reported symptoms, which may introduce gaps in understanding and hinder the establishment of comprehensive clinical profiles, integrating objective data through multimodal assessments is essential. Combining neuroimaging, serological testing, and advanced cognitive evaluation tools could enhance diagnostic precision and mechanistic insights. Large-scale international research initiatives and longitudinal studies are also needed to enhance the generalizability of findings and capture the long-term evolution of CI in NADs. Standardizing methodologies across diverse populations would not only strengthen the clarity and rigor of research but also facilitate the identification of modifiable risk factors and new therapeutic targets.

Ultimately, these steps may pave the way toward a more precise understanding of gender-based cognitive differences in NADs and beyond, while promoting integrative approaches to chronic disease management, and support the development of more equitable, evidence-based healthcare strategies tailored to diverse populations.

## Data Availability

The original contributions presented in the study are included in the article/Supplementary material, further inquiries can be directed to the corresponding author.

## Glossary

AITD: autoimmune thyroid disease
ASD: autism spectrum disorder
ATD: anti-thyroid drug
BBB: blood–brain barrier
BDI-II: Beck’s Depression Inventory Second Edition
BDNF: brain-derived neurotropic factor
BMI: body mass index
CFS: chronic fatigue syndrome
CI: cognitive impairment
CIS: clinically isolated syndrome
CNS: central nervous system
CSF: cerebrospinal fluid
DMN: default mode network
DMTs: disease-modifying therapies
EDS: excessive daytime sleepiness
EDSS: Expanded Disability Status Scale
FMS: fibromyalgia syndrome
FSH: follicle-stimulating hormone
GABA: gamma-aminobutyric acid
GBS: Guillain-Barré syndrome
GD: Graves’ disease
GH: growth hormone
GM: gray matter
HPA axis: hypothalamic–pituitary–adrenal axis
HPT: hypothalamic–pituitary-thyroid
HR: heart rate
HRQoL: health-related quality of life
HT: Hashimoto’s thyroiditis
ICU: intensive care unit
IL-1β: interleukin-1 beta
IL-6: interleukin 6
IVIG: intravenous immunoglobulin
LH: luteinizing hormone
LOS: length of stay
MG: myasthenia gravis
MG-ADL: MG activities of daily living
MG-QoL15: Myasthenia gravis-specific 15-item Quality of Life scale
MRI: magnetic resonance imaging
MS: multiple sclerosis
MT: molecular therapy
NAA: N-acetyl-aspartate
NADs: neurological auto-immune disorders
NF-κB: nuclear factor-kappa B
NOS: Newcastle-Ottawa Scale
NREM: non-rapid eye movement
NT1: narcolepsy type 1
PASAT: Paced Auditory Serial Audition
PCC: patient-centered care
PICS: post-intensive care syndrome
PPMS: Primary progressive multiple sclerosis
PRISMA: Preferred Reporting Items for Systematic Reviews and Meta-Analysis
QMG: Quantitative Myasthenia Gravis Score
QoL: quality of life
RCT: randomized controlled trial
REM: rapid eye movement
RoB: risk of bias
ROB2: Cochrane Risk of Bias 2.0
RRMS: relapsing–remitting multiple sclerosis
SF-36: Short-Form Health Status
SIK1: salt-inducible kinase 1
SREAT: steroid-responsive encephalopathy
TED: thyroid eye disease
ThyPRO: Thyroid-Related Patient-Reported Outcome
TIAs: transient ischemic attacks
TNF-*α*: Tumor necrosis factor alpha
ToM: theory of mind
TPC: tender point count
