# Supplementary material for: Gender disparities in cognitive impairment across neurological autoimmune disorders: a systematic review
Source: Front Neurol. 2025 Apr 23;16:1555407. doi: 10.3389/fneur.2025.1555407 (PMC12055792; doi:10.3389/fneur.2025.1555407)
Supplement: Supplementary file 1 [file Table_1.DOCX]

Supplementary Material

# Table 1 – Gender Disparities of CI across NADs_Summary of Findings

| **Condition/ NAD** | **Title & DOI** | **Author(s), Year** | **Study Design** | **Population** | **Main Findings** | **Quality/ Risk of Bias (Tool Used)** |
| --- | --- | --- | --- | --- | --- | --- |
| **Hashimoto's Thyroiditis (HT)** | No studies found. | N/A | N/A | N/A | N/A | N/A |
| **Graves' Disease (GD)** | Long-Term Outcome of Graves' Disease: A Gender Perspective. DOI: 10.1089/whr.2023.0073 | Calissendorff et al. 2023 | Prospective cohort study (Longitudinal Follow-Up; 6–10 years). | N: 1186 GD patients Gender: 973 women (82%), 213 men (18%) Age: Mean 47 (±14) years (women), 49 (±14) years (men) Ethnicity/Country: Sweden | - Short-Form Health Status (SF-36): Women have worse bodily pain scores. - Thyroid-Related Patient-Reported Outcome (ThyPRO): Women have higher depression (p<0.05), impaired sex life (p<0.05), and cosmetic complaints (p<0.05). - Hypothyroidism: 2× higher prevalence in women (29.5% vs. 14.9% in men, p<0.05) \| accordingly Levothyroxine use: Women twice as likely to require it post-antithyroid drugs (ATD) (p<0.05). - Feeling of recovery: No gender difference, but levothyroxine users (33.2%) felt less recovered vs. non-users (13.9%). | Tool: Newcastle-Ottawa Scale (NOS) for cohort studies.  Strengths: large cohort, long follow-up (6–10 years), validated assessment tools, adjustment for age/comorbidity.  Limitations: observational design; possible confounding (menopausal status, biochemical thresholds for levothyroxine, thyroid antibody levels), selection bias (60% response rate), self-report bias.  Risk of Bias: Moderate. |
| **Fibromyalgia (FMS)** | Sex-Related Differences in Symptoms and Psychosocial Outcomes in Patients With Fibromyalgia: A Prospective Questionnaire Study. DOI:10.1016/j.mayocpiqo.2020.06.009 | Jiang et al. 2020 | Prospective cohort questionnaire study. | N: 668 patients Gender: 606 women (90.7%), 62 men (9.3%) Age: Mean 47.2±13.0 years Ethnicity/Country: 93.5% White men, 88.6% White women; USA (the Fibromyalgia and Chronic Fatigue Clinic). | - Higher tender point count (TPC) in women (p<0.001). - No sex differences in depression, anxiety, sleep problems, FMS symptom severity, cognitive dysfunction, or quality of life (QoL), after Benjamini-Hochberg adjustment. - Lower symptom severity in women (p=0.03) pre-adjustment; (Revised Fibromyalgia Impact Questionnaire). | Tool: ROBINS-I (Risk Of Bias In Non-randomized Studies). - Strengths: relatively large cohort, validated questionnaires, adjusted for confounders (age, BMI, education). - Limitations: selection bias (single-center; limited generalizability), self-report bias (subjective symptom reporting), non-balanced gender representation. Risk of Bias: Moderate. |
|  | Gender Differences in Symptoms, Health-Related Quality of Life, Sleep Quality, Mental Health, Cognitive Performance, Pain-Cognition, and Positive Health in Spanish Fibromyalgia Individuals: The Al-Ándalus Project. DOI:10.1155/2016/5135176 | Segura-Jiménez et al. 2016 | Cross-sectional study | N: 652 participants (405 FMS patients, 247 healthy controls) Gender: FMS patients (384 women, 21 men), healthy controls (195 women, 52 men) Age: Mean ~49 years (FMS women), ~47 years (FMS men) Ethnicity/Country: Spain (Andalusia). | FMS Group: - Men had better working memory (PASAT; p<0.01). - Women had shorter sleep latency (47.9 vs. 72.7 mins, p=0.013). Healthy controls non-FMS Group: - Men had higher pain thresholds (all tender points except epicondyles, p<0.01), and better working memory than women (p<0,01). - Women had better verbal memory (RAVLT, P ≤ 0.01). | Tool: ROBINS-I. - Strengths: relatively large sample, comprehensive assessments using validated tools, adjusted for confounders. - Limitations: cross-sectional design (no causality), small male subgroup, self-report bias. Risk of Bias: Moderate. |
|  | Gender influence on clinical manifestations, depressive symptoms and brain-derived neurotrophic factor (BDNF) serum levels in patients affected by fibromyalgia. DOI:10.1007/s10067-022-06133-y | Iannuccelli et al. 2022 | Cross-sectional study | N: 241 participants (201 FMS patients, 40 healthy controls) Gender: Patients (172 women, 29 men) matched by age/sex with the healthy controls Age: Mean 49 years (FMS group) Ethnicity/Country: Italy (Sapienza University of Rome). | - FMS Women: Higher pain, fatigue, memory issues, tenderness, balance problems and sensitivity to environmental stimuli compared to males. - FMS Men: Higher depressive symptoms (72.4% vs. 51.9%, p=0.038). - BDNF: Lower in FMS patients compared to healthy controls (p<0.0001); FMS men had lower BDNF than FMS women and than healthy control men, FMS women had lower BDNF than healthy control women. | Tool: ROBINS-I. - Strengths: validated tools employed, BDNF measured via ELISA with sensitivity controls, adjusted for confounders (age, BMI). - Limitations: small male subgroup, single-center recruitment (selection bias), cross-sectional design (no causality). Risk of Bias: Moderate. |
| **Guillain-Barré Syndrome (GBS)** | Factors associated with long-term functional outcomes and psychological sequelae in Guillain–Barre syndrome. DOI: 10.1007/s00415-010-5653-x | Khan et al. 2010 | Prospective observational cohort study with structured interviews and medical record review. | N: 76 (from 157 initially identified; 62% recruitment rate) Gender: 60% males, 40% females Age: Mean 56 years  Ethnicity/Country: Australia (Royal Melbourne Hospital) | - Demographic: Females and older adults (57+ years) had worse functional (↓FIM scores) and psychological outcomes (↑DASS scores). - Clinical severity indicators: ICU admission, prolonged acute hospital length of stay (LOS) >11 days, and discharge destination (to a rehabilitation facility) linked to lower function and higher participation restrictions. - Time since diagnosis showed no association with outcomes of functional independence or psychological wellbeing. - Overall, a high percentage of participants reported moderate, severe or extreme levels of depression, anxiety and stress. | Tool: Newcastle-Ottawa Scale (NOS). - Strengths: validated tools used, comprehensive assessments. - Limitations: small sample, regional bias, self-reporting bias, no adjustment for confounders. Risk of Bias: Moderate. |
| **Myasthenia Gravis (MG)** | Gender differences in quality of life among patients with myasthenia gravis in China. DOI: 10.1186/s12955-020-01549-z | Dong et al. 2020 | Cross-sectional study (using online questionnaires). | N: 1815 patients (from 2000 initially recruited) Gender: 1194 females (66%), 621 males (34%) Age: Mean 43.4 years (males), 39.8 years (females) Ethnicity/Country: China | - Gender Disparity: Females reported lower HRQoL across all domains (physical, social, and emotional; p < 0.05). - Comorbidities: Interaction between gender and comorbidities worsened HRQoL more in females (p < 0.05). - Key Factors: Unemployment (p < 0.001), MG exacerbations (p < 0.001), and active lifestyle (p < 0.001) strongly influenced HRQoL. - Clinical: Females had worse MG activities of daily living scale (MG-ADL) scores and higher rates of autoimmune thyroid disease. | Tool: JBI Critical Appraisal Checklist for Analytical Cross-Sectional Studies. - Strengths: validated tools used, comprehensive assessments, adjustment for confounders. - Limitations: convenience sampling, self-reporting bias, no objective clinical severity measures, lack of causality due to study design. Risk of Bias: Moderate. |
|  | Female sex and overweight are associated with a lower quality of life in patients with myasthenia gravis: a single center cohort study. DOI: 10.1186/s12883-023-03406-0 | Wilcke et al. 2023 | Mixed retrospective-prospective cohort study (monocentric). | N: 165 patients. Gender: 85 males (51.5%) , 80 females (48.5%). Age: Mean 59.8 years at study entry; females younger at symptom onset (50.1 vs. 58.4 years). Ethnicity/Country: Germany. | - Gender: Females had lower HRQoL (MG-QoL15) and worse functional status (MG-ADL). - BMI: Higher BMI correlated with poorer HRQoL (β = +1.29 per BMI unit, p = 0.005) and MG-ADL (p = 0.017). - Clinical: Disease severity (QMG) strongly impacted HRQoL (β = +17.6, p < 0.001). Females experienced longer diagnosis delays (2.15 vs. 0.87 years, p = 0.034). - Comorbidities: Females had higher rates of depression (28.7% vs. 15.3%, p = 0.04) and autoimmune diseases (27.5% vs. 9.4%, p = 0.004). | Tool: JBI Critical Appraisal Checklist for Analytical Cross-Sectional Studies. - Strengths: validated tools used, comprehensive assessments, adjustment for confounders (age, disease severity). - Limitations: small sample for BMI analysis, retrospective design, convenience sampling. Risk of Bias: Moderate. |
| **Multiple Sclerosis (MS)** | Can we predict cognitive decline after initial diagnosis of multiple sclerosis? Results from the German National early MS cohort (KKNMS). DOI: 10.1007/s00415-018-9142-y | Johnen et al. 2019 | Prospective multicenter cohort study (NationMS), longitudinal (baseline and 1-year follow-up). Bayesian multilevel regression models. | N: 1123 patients; newly diagnosed multiple sclerosis (MS) or clinically isolated syndrome (CIS). Gender: 2.2:1 female-to-male ratio. Age: Median of onset is 31,71 years. Ethnicity/Country: Germany. | - 22% of patients had CI at baseline (processing speed/executive function most affected).  - Baseline CI predictors: Older age, male sex, lower education, higher physical disability (EDSS), and depression (BDI-II) linked to greater CI severity.  - Demographic, clinical, and conventional MRI data insufficient for identifying short-term cognitive change risks in newly diagnosed MS or CIS patients. | Tool: Newcastle-Ottawa Scale (NOS). - Strengths: large multicenter cohort, standardized protocols, controlled for age/gender/education. - Limitations: short follow-up, unmeasured confounders (cognitive reserve), practice effects in cognitive testing. Risk of Bias: Moderate. |
|  | Sex Differences in Resting-State Functional Connectivity in Multiple Sclerosis. DOI: 10.3174/ajnr.A3630 | Koenig et al. 2013 | Cross-sectional case-control study. | N: 64 participants; 32 MS patients matched with 32 healthy controls.  Gender: MS patients (16 males, 16 females), healthy controls (16 males, 16 females).  Age: Mean age of 41.85 years.  Ethnicity/Country: USA. | - MS patients showed stronger connectivity from posterior cingulate to medial frontal gyri, anterior cingulate, right putamen, and left middle temporal gyrus compared to healthy controls (P < .0005).  - Male MS patients had weaker connectivity to the caudate compared to female MS patients (P = .004). While male controls have stronger connectivity between posterior cingulate and left prefrontal cortex than female controls. - Female MS patients had stronger connectivity between posterior cingulate and left prefrontal cortex compared to female controls. | Tool: Newcastle-Ottawa Scale (NOS). - Strengths: Age/sex matching of controls, standardized MRI protocols and motion correction, blinded neuropsychological assessments. - Limitations: small sample size,cingle-center design (potential selection bias), lack of longitudinal data to assess causality.  Risk of Bias: Moderate. |
|  | What Can We Learn from Sex Differences in MS? DOI: 10.3390/jpm11101006 | Coyle et al. 2021 | Integrative Literature review. | N/A (discusses global MS demographics). | - MS is more common in females, with high variability. - Relapsing MS is the most common phenotype (RRMS). - PPMS has a later age of onset (decade later than RRMS). - MS disease activity decreases during pregnancy (3rd trimester), rebounds postpartum, and may worsen after menopause. - Males more likely to develop primary progressive MS (PPMS), with worse prognosis, cognitive impairment, and disability. - Males with RRMS show more motor issues and fewer optic neuritis cases. - MS males have lower LH, FSH, and testosterone levels. - Sex hormones (estriol, testosterone) have immunomodulatory effects on MS.  - Progressive MS males show more severe HPT axis abnormalities than RRMS males. - Obesity and high BMI in adolescence increase MS risk in males. - Neuroimaging: Greater gray matter atrophy in males; hormonal influences on CNS structure. - Comorbidities: Higher vascular comorbidities in males; mental health issues in females. - Treatment: Safety considerations for disease-modifying therapies (DMTs) during pregnancy and breastfeeding. | Tool: SANRA (Scale for the Assessment of Narrative Review Articles). - Strengths: comprehensive scope and up-to-date coverage of data, clinically relevant insights (pregnancy, DMT use). - Limitations: methodological limitations (literature review). Risk of Bias: Moderate. |
|  | Potential biological contributers to the sex difference in multiple sclerosis progression. DOI: 10.3389/fimmu.2023.1175874 | Alvarez-Sanchez et al. 2023 | Integrative Literature review. | N/A (synthesizing human and animal studies, with diverse demographics) | - Males exhibit faster progression, more cortical lesions, and cognitive decline (verbal and non-verbal memory, information processing speed, attention, and executive functioning deficits) than females.  - Males exhibit lower functional connectivity and network efficiency.  - Females show better remyelination, robust anti-viral responses, and hormonal neuroprotection.  - These gender-related disparities related to numerous factors (including immune, hormonal, structural, environmental, metabolic, and gastrointestinal). | Tool: SANRA. - Strengths: comprehensive coverage of existing studies; highlights understudied mechanisms. - Limitations: methodological limitations (literature review). Risk of Bias: Moderate. |
|  | Quantitative effect of sex on disease activity and disability accumulation in multiple sclerosis. DOI: 10.1136/jnnp-2022-328994 | Magyari et al. 2022 | Observational cohort study (Nationwide population-based). | N: 9647 patients; RRMS treated with DMTs (since 1996). Gender: 3028 males, 6619 females. Age: Mean age at onset is 34.3 (male), 33.5 (females). Ethnicity/Country: Danish population (Denmark). | - Women had 16% higher relapse rates than men, but this difference disappeared after age 50. - Men showed faster disability accumulation (annual EDSS increase: 0.07 vs. 0.05 in women, p = 0.017). - Men had higher hazards of reaching EDSS 4 (HR: 1.34) and EDSS 6 (HR: 1.43) (p < 0.001). - Gender-related differences in neurodegenerative symptoms became pronounced after age 45. | Tool: ROBINS-I. - Strengths: nationwide registry data minimizing selection bias, adjustment for confounders, large sample size. - Limitations: exclusion of untreated/progressive MS patients, lack of MRI data, potential unmeasured confounders (lifestyle factors). Risk of Bias: Moderate. |
|  | Estriol-mediated neuroprotection in multiple sclerosis localized by voxel-based morphometry. DOI: 10.1002/brb3.1086 | MacKenzie-Graham et al. 2018 | Randomized Controlled Trial (Phase 2) “A Combination Trial of Copaxone plus Estriol in RRMS” | N: 111 RRMS patients (62 estriol-treated, 49 placebo-treated).  - Gender: Female.  - Age: 18–50 years (mean 37.3 ± 7.5).  - Ethnicity/Country: 81.1% Caucasian, 9.9% Black, 8.1% Hispanic; USA-based. | - Estriol preserved gray matter (GM) in frontal and parietal cortices, correlating with improved PASAT scores.  - Estriol group had less whole GM atrophy (0.5% vs. 1.5% annualized loss).  - Early intervention with a neuroprotective agents is better to prevent GM loss and cognitive impairment (CI).  - No significant effects on motor outcomes. | Tool: Cochrane Risk of Bias 2.0 (RoB 2) . - Strengths: randomized, blinded outcome assessment, controlled for multiple comparisons. - Limitations: small sample size, multi-site MRI variability, potential practice effects on PASAT. Risk of Bias: Moderate. |
| **Narcolepsy Type 1 (NT1)** | Gender medicine and sleep disorders: from basic science to clinical research. DOI: 10.3389/fneur.2024.1392489 | Perger et al. 2024 | Integrative literature review. | N/A (depends on each study included; some studies are not accessible). | Females exhibit an earlier onset of EDS and cataplexy, more severe EDS, and longer diagnostic delay compared to males. Males experienced fewer nocturnal awakenings compared to females. Neurobiological differences suggest sex-specific variations in narcolepsy symptoms and sleep patterns. Findings underscore the need for more systematic studies to confirm sex-related disparities and their mechanisms. | Tool: SANRA. - Strengths: comprehensive Scope, multidisciplinary analysis, balanced synthesis (acknowledges conflicting data and methodological heterogeneity), clinical relevance. - Limitations: narrative design limitations, geographic bias (European cohorts), confounding variables. Risk of Bias: Moderate. |

**
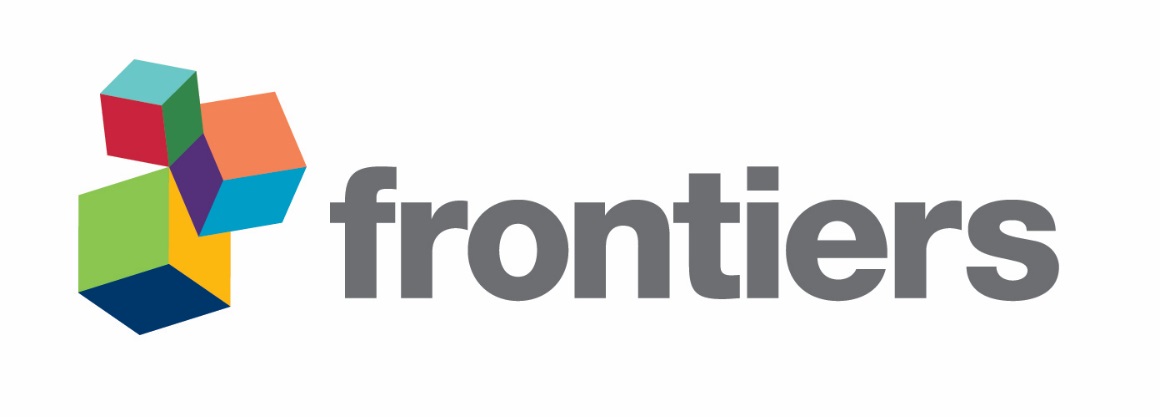
**
