# Supplementary material for: Gender disparities in cognitive impairment across neurological autoimmune disorders: a systematic review
Source: Front Neurol. 2025 Apr 23;16:1555407. doi: 10.3389/fneur.2025.1555407 (PMC12055792; doi:10.3389/fneur.2025.1555407)
Supplement: Supplementary file 2 [file Table_2.DOCX]

Supplementary Material

# Table 2 – Summary & Interpretations of the Retrieved Studies on Gender Disparities of CI across NADs

| **Condition/ NAD** | **Author(s) & Year** | **Synthesis of The Findings** | **Interpretations & Implications of The Findings** |
| --- | --- | --- | --- |
| **Hashimoto's Thyroiditis (HT)** | N/A | N/A | Research gaps:  - Cognitive health is a secondary concern in HT research.  - Gender-related disparities in CI are further overlooked. |
| **Graves' Disease (GD)** | Calissendorff et al. 2023 | Women with GD experience worse long-term outcomes, including doubled hypothyroidism rates (post-treatment), persistent depression, bodily pain, and cosmetic complaints. | Research Gaps:  - Limited data availability, with a single study insufficient to draw strong conclusions.  - Cognitive health is not directly assessed; existing research focuses on GD-related poor quality of life (QoL) rather than cognitive outcomes.  - Causality remains unproven due to various confounding factors (menopause, psychosocial influences, etc..). |
| **Fibromyalgia (FMS)** | Jiang et al. 2020 | Women with FM exhibit higher tender point counts, but no significant sex differences exist in mood disorders, sleep, fatigue, cognitive functioning, or QoL. | Similarities across studies: - Higher pain-related symptoms in women (tender points, pain severity) compared to men. - Need for gender-inclusive research (gaps in male representation and call for larger studies).  Inconsistencies & Gaps: - Contradictory mood/cognitive findings. - Sleep latency: Only the 2nd study highlighted that women's shorter sleep latency. - Biomarker potential but insufficient data: BDNF’s role remains unexplored. - Lack of standardized research protocols. |
|  | Segura-Jiménez et al. 2016 | FMS men exhibit better working memory and higher pain thresholds than FMS women, while FMS women report shorter sleep latency. Gender-specific differences in FMS symptoms are not consistent, and larger studies with better male representation are needed. |  |
|  | Iannuccelli et al. 2022 | Women with FMS report more pain-related symptoms, while men exhibit higher depressive symptoms. BDNF is significantly lower in FMS patients compared to healthy controls, showing potential as a diagnostic biomarker. Gender-specific symptom profiles highlight the need for tailored diagnostic approaches. |  |
| **Guillain-Barré Syndrome (GBS)** | Khan et al. 2010 | GBS-related psychological burdens (stress, depression, anxiety) are long-term outcomes that need to be acknowledged and managed. High-risk subgroups include females, older adults, ICU patients, and those requiring prolonged hospitalization. | Research gaps: - Data scarcity; a single study found. - Indirect assessment of cognitive dysfunction (based on correlations between psychological burdens and cognitive function). - Small sample size poorly representing the GBS community, and limiting generalizability. |
| **Myasthenia Gravis (MG)** | Dong et al. 2020 | Gender differences in HRQoL among MG patients are mediated by comorbidities, employment status, and disease exacerbations. Females require targeted management of comorbidities and psychosocial support to improve HRQoL. | Similarities across studies:  - Female sex as a significant predictor of reduced health-related quality of life (HRQoL) in MG patients.  - Call for gender-specific approaches for MG management.  Inconsistencies & Gaps:  - Higher BMI (alongside female sex) as a key predictor of reduced HRQoL.  - Weight management as a critical component of care, distinct from Study 1’s focus on psychosocial factors.  - Limited scope of investigations.  - Cognitive Impairment is not directly assessed. |
|  | Wilcke et al. 2023 | Female sex and higher BMI are significant predictors of reduced HRQoL in MG. Gender-specific medicine and weight management are critical for improving outcomes. |  |
| **Multiple Sclerosis (MS)** | Johnen et al. 2019 | Routine clinical/MRI data predict baseline CI but not short-term decline. Improved cognitive tests and advanced biomarkers are needed for progression prediction. | Similarities across studies:  - Males exhibit worse prognoses, faster disability progression, and higher rates of cognitive decline.  - Relapse rates vary by gender and age, with a higher prevalence in females before menopause (hormonal influences).  - Sex hormones (estriol, testosterone) modulate immune activity and provide neuroprotective effects.  - The severity of CI is influenced by multiple factors, primarily older age, male sex, and psychosocial and economic variables.  - Structural differences, such as grey matter (GM) atrophy and connectivity patterns, correlate with sex-specific cognitive outcomes, with women generally exhibiting better connectivity.  Inconsistencies & Gaps:  - A lack of integrative investigations, with many studies highlighting individual factors without efforts to enhance generalizability.  - Understudied populations, as some research focuses solely on specific multiple sclerosis (MS) subtypes and without considering ethnic diversity. |
|  | Koenig et al. 2013 | Sex and disease status interact to produce differences in functional connectivity patterns. Researchers must account for sex differences in MS neuroimaging studies. |  |
|  | Coyle et al. 2021 | Sex differences significantly impact MS epidemiology, clinical course, and therapeutic approaches. Research on sex-specific mechanisms may lead to personalized treatments and improved clinical guidelines. |  |
|  | Alvarez-Sanchez et al. 2023 | Sex differences in MS progression are driven by biological mechanisms including immune cell reactivity, hormonal influences, iron metabolism, and neuronal vulnerability. |  |
|  | Magyari et al. 2022 | Sex hormones likely drive inflammatory activity differences in younger patients, while men are more vulnerable to neurodegeneration post-age 45. Findings support personalized MS management by sex. |  |
|  | MacKenzie-Graham et al. 2018 | Estriol may protect GM in cognition-related regions, supporting its role as a neuroprotective therapy. |  |
| **Narcolepsy Type 1 (NT1)** | Perger et al. 2024 | The study underscores gender disparities in NT1 symptomology and outcomes, highlighting cognitive disparities indirectly. There is a need for standardized, globally inclusive research to further explore and validate these findings. | Research Gaps:  - Limited data availability, with only a single narrative study combining animal and human data, which contains significant shortcomings and missing information (confounding factors, mechanistic insights, etc..).  - Research blindspots: (1) the autoimmune nature of NT1 has only recently been recognized, (2) the role of hypocretin remains debated, and (3) NT1 is primarily studied within the broader category of sleep disorders. |

**
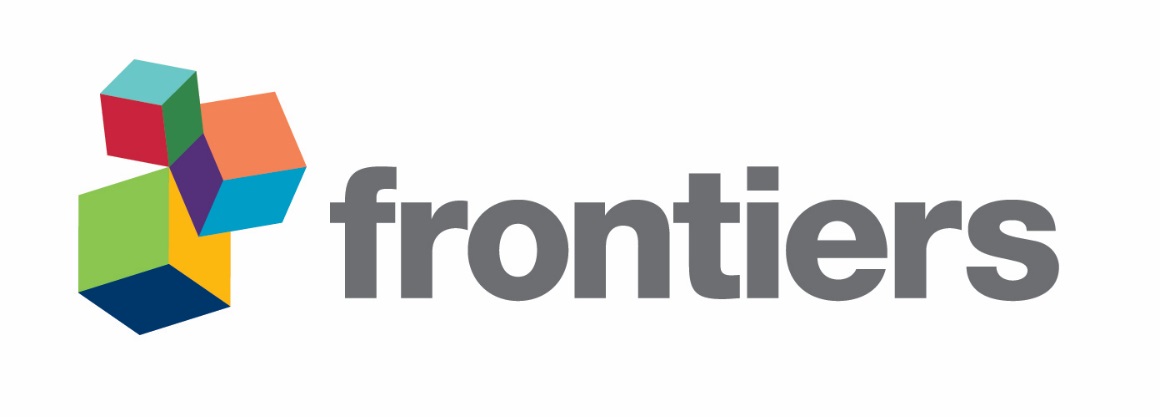
**
